# Turning back time: effects of young plasma on pan epigenetic clocks and implications for the heart

**DOI:** 10.20517/jca.2023.44

**Published:** 2023-12-31

**Authors:** Kathleen C. Woulfe, Emma L. Robinson

**Affiliations:** 1 Department of Medicine, Division of Cardiology, University of Colorado Anschutz Medical Campus, Aurora, CO 80045-2507, USA.; 2 Consortium for Fibrosis Research & Translation, University of Colorado Anschutz Medical Campus, Aurora, CO 80045-2507, USA.

Mammalian aging is a consequence of time, but the inevitability of aging is contested. Chronological aging (based on the time from birth to current age) is distinct from biological aging, which is thought to be based on the accumulation of cellular damage, and has been associated with a number of interconnected “hallmarks”. Since the first description of these “hallmarks of aging”, research efforts have focused on determining whether experimental manipulation of these signatures of aging can affect lifespan and healthspan. To date, 12 hallmarks of aging have been proposed: genomic instability, telomere attrition, epigenetic alterations, loss of proteostasis, disabled macroautophagy, deregulated nutrient-sensing, mitochondrial dysfunction, cellular senescence, stem cell exhaustion, altered intercellular communication, chronic inflammation, and dysbiosis^[1]^. Notably, complex degenerative diseases are ultimately caused by progressive changes wrought by one or more cellular perturbations known as a hallmark of aging in critical organs. These include many cancers, cardiovascular and cerebrovascular diseases, and neurodegeneration, which, without a doubt, comprise the greatest socio-economic burdens of the 21st century.

A wealth of studies have demonstrated a connection between not only chronological but also biological aging of different mammalian tissues according to their DNA methylome. Within gene bodies, DNA methylation (DNAme) broadly promotes gene transcription. Within gene promoter regions, DNAME is associated with transcriptional repression and is abundant in gene-poor and silenced heterochromatic genomic regions. Loss of DNAme can cause transcriptional dysregulation and loss of genomic integrity. Gradual loss of DNAme with progressive rounds of DNA replication is linked to cellular senescence and exhaustion of stem cell populations. Other than the passive loss of DNA through replicative cycles, DNA methylation can be lost actively through oxidation reactions mediated by Ten-Eleven Translocase (TET) enzymes. TET1/2/3 convert DNAme first to hydroxymethylation (DNAhme), followed by a series of further processing to remove methylation. Mutations in TET2 and DNA methyltransferase 3A have been linked with age-associated clonal hematopoiesis atherosclerosis, inflammation, and worsened cardiovascular outcomes^[2]^. Epigenetic age has been shown to predict cardiovascular and cardiorespiratory health outcomes, as well as heart failure risk following cardiotoxic chemotherapy treatments^[3]^.

In 2013, Steven Horvath and team published a landmark paper presenting a DNAme clock, where the knowledge of sites and abundance of CpG DNAme in different healthy human tissues and cells of known chronological age were used to generate a reference set of DNA methylome profiles of tissues^[4]^. This highly-validated, mathematically-derived epigenetic clock can be used to estimate the chronological age of test tissues. The DNAme clock can also be used to assess the biological age of tissues of known age, compared with reference tissues of the same chronological age.

Importantly, the fact that cellular attributes that characterize aging (including epigenetic alterations) are highly signal-responsive and regulated by their extracellular environment provides clear evidence that biological age is modifiable. For the past 70 years, it has been known that exposing older cells and animals to a “younger” blood-based secretome (heterochronic parabiosis) has a profound effect on reversing age-associated gene expression and cellular hallmarks of aging^[5]^.

A recent paper published in *Geroscience* by Horvath *et al*. demonstrated that treating old rats with an extracellular vesicle (EV)-enriched fraction of plasma isolated from young pigs substantially modifies the epigenetic age of the old rats^[6]^. By establishing new epigenetic clocks from DNAme profiles of all rat tissues, and a human/rat clock, this study specifically demonstrates that the EV-enriched fraction of young plasma fraction isolated from a different species can modulate DNAme in tissues of aged recipient rats. Impressively, the 2-dose treatment regimen of EVs isolated from young pigs significantly reduced the epigenetic age of the treated animals based on six different clocks. Additionally, a decrease in markers of inflammation and oxidative stress was observed in the old rats that received the EV-enriched young plasma [Figure 1]. This study validates and extends previous work in mice, as well as showing that cross-species treatment holds promise.

While pan-tissue epigenetic clocks established in this study show significantly accurate age estimation in individual tissues, such as blood, liver, and hypothalamus, predictive power was lower for the heart (e.g., Liver R > 0.95 *vs*. Heart R > 0.89). This is particularly interesting given that the clocks were trained in both male and female tissues. Notably, it has been reported that when considering heart function and increased markers of aging, such as the frailty assay developed by Dr. Feridooni *et al.*, male mice have a high degree of association between increased frailty score and decreased cardiac function^[7]^. This relationship is far less robust in female animals. These findings implicate that systemic aging factors that contribute to cardiac functional decline may be distinct in males and females. In the case of this study, it may be important to develop sex-specific cardiac clocks and determine if epigenetic changes in the heart have sexually dimorphic characteristics.

Horvath *et al.* report that the EV-enriched plasma treatment decreased the epigenetic age of the heart by 46%^[6]^. Impressively, old animals treated with the young EV-enriched fraction demonstrated a significant reduction in oxidative stress in heart tissue. Even though this study did not measure cardiac function, we are able to extrapolate potential benefits based on other studies reporting direct cardiac benefits in heterochronic parabiosis and the use of young EVs. Assessment of cardiac function in an old mouse paired with a young mouse in heterochronic parabiosis for 4 weeks has been shown to decrease cardiac hypertrophy, cardiomyocyte size, and gene expression of BNP and ANF, which are factors that increase under conditions of cardiac dysfunction. It has also been reported that EVs isolated from cardiosphere-derived cells, when injected monthly for 4 months into old rats, decrease heart weight to body weight ratio, cardiomyocyte size, BNP, and cardiac fibrosis. Other known benefits associated with factors in young blood include the enhancement of endothelial vasorelaxation and aortic responsiveness to acetylcholine signaling. The improvement in vascular function not only decreases the afterload the heart encounters during pumping but also delivers more blood to the cardiac tissue, thereby contributing to cardiac health.

Taken together, these studies indicate that specific cargo in the EVs is unique in young animals and can mediate adverse cardiac remodeling associated with increased age. Horvath *et al.* demonstrated that longnon-coding RNAs (lncRNAs) are highly abundant within the EV fraction from young pig blood^[6]^. It is unlikely that heterochronic parabiosis, in its current form, will become a rejuvenation remedy in humans; however, the identification of critical differences in circulating factors from young subjects may provide the potential for therapeutics. LncRNAs, for example, present an intriguing arena as therapeutic modalities and targets. These relatively newly discovered biological molecules are more numerous in the human genome than protein-coding genes and have been linked with almost every hallmark of aging, including cellular senescence, mitochondrial dysfunction, intercellular communication, epigenetic alterations, and genome integrity and stability. Pathological aging-associated remodeling of the heart, including cardiac hypertrophy, fibrosis, inflammation, endothelial cell dysfunction, and stress-associated cardiac gene signatures, have all been reported to be under the regulation of lncRNAs *in vitro* and *in vivo*. Deep sequencing of the EV content from pig plasma yielding the most potent anti-aging effects when administered through parabiosis will provide a new tool kit of molecules and potential therapeutic targets to specifically reverse hallmarks of aging in vital organs, including the heart.

A major hurdle in the field of academic and clinical geroscience is that aging is presently not officially classified as a “disease”. Clinical trials evaluating the efficacy of new pharmacological compounds to modify hallmarks of aging in the United States are not performed and pose practical considerations, restricting most interventional studies in chronobiology to pre-clinical and cellular models. Exceptions are nonpharmacological approaches such as caloric restriction and intermittent fasting, both of which have shown variable amelioration in surrogate biomarkers of aging. With evidence supporting epigenetic alterations as a central mediator of a number of other hallmarks of aging, epigenetic drugs pose an intriguing therapy against aging and aging-associated diseases. Indeed, histone deacetylase and DNMT inhibitors are already on the market for various cancers. Potent pharmacological TET inhibitors have only recently been developed and are yet to be tested chronically *in vivo*.

DNAme and DNAhme also exist on the mitochondrial genome (mtDNA). While comprising relatively few genes compared with the nuclear genome (37 *vs.* approx. 20,000 protein-coding genes), with mitochondrial dysfunction being one of the original hallmarks, it will be interesting to examine how aging affects mtDNA DNA methylation and whether this corresponds with chronological, replicative or biological aging. Altered mtDNAme and mtDNAhme with aging have been noted in the brain tissue of mice^[8]^.

Overall, narrowing down the mechanisms behind how treating an old animal with EVs from a young animal can *turn back time* on epigenetic clocks may provide novel targets that can modulate biological aging and improve health and lifespan. The intersection of factors that modify epigenetic clocks and how that impacts organ function will expand our understanding of specific contributors that change the trajectory of aging.

## Figures and Tables

**Figure 1.**
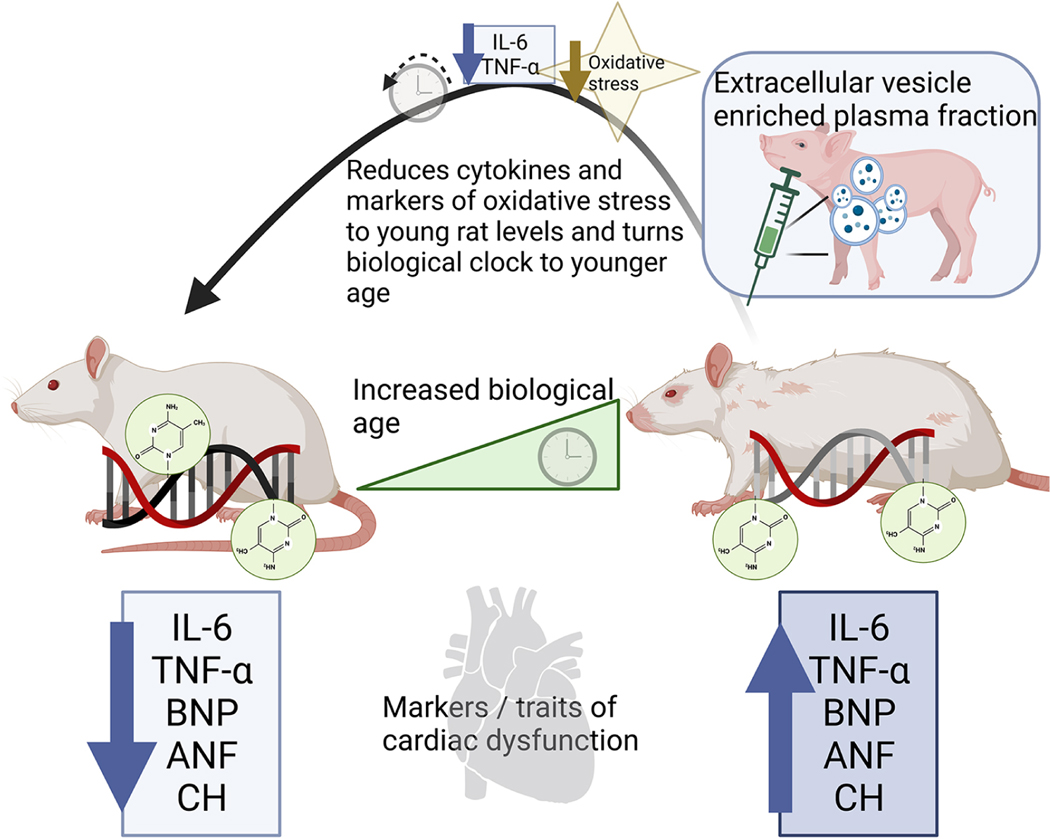
Heterochronic parabiotic treatment of old rats with the extracellular vesicle-enriched fraction of young pig plasma *turns back* the epigenetic clock in aged rat tissues, and decreases cytokines and oxidative stress. IL-6: Interleukin 6; TNF-α: tumor necrosis factor-alpha; BNP: brain natriuretic peptide; ANF: atrial natriuretic factor; CH: cardiac hypertrophy.
